# Systematic evaluation of MAIT and iNKT abundance as checkpoint blockade biomarkers

**DOI:** 10.3389/fimmu.2026.1917317

**Published:** 2026-09-15

**Authors:** Yueyi Li, LinLin Chen, Shiwen Shan, Haiyang Wang, Xiaofang Xing, Jian Zhang

**Affiliations:** 1 Academy of Medical Engineering and Translational Medicine, Tianjin University, Tianjin, China; 2 State Key Laboratory of Advanced Medical Materials and Devices, Tianjin, China; 3 Key Laboratory of Systems Bioengineering (Ministry of Education), Tianjin University, Tianjin, China; 4 Beijing Institute of Radiation Medicine, Beijing, China; 5 Department of Gastrointestinal Translational Research, Key Laboratory of Carcinogenesis and Translational Research (Ministry of Education), Peking University Cancer Hospital, Beijing, China

**Keywords:** immune checkpoint blockade (ICB), invariant natural killer T (iNKT) cells, mucosal-associated invariant T (MAIT) cells, T cell receptor (TCR), unconventional T cells

## Abstract

**Introduction:**

MAIT and iNKT cells are unconventional T cell lineages that bridge innate and adaptive immunity and have been implicated in antitumor immune responses and immunotherapy outcomes. However, evidence to date has largely derived from individual studies of selected cancer types or specialized immune profiling cohorts. Whether MAIT and iNKT abundance provides reproducible clinical information across large, multi-cancer immune checkpoint blockade (ICB) cohorts remains insufficiently evaluated.

**Methods:**

We applied an AI-based TCRβ inference framework to estimate MAIT and iNKT relative abundance from bulk TCRβ repertoires in healthy controls, patients with cancer, and ICB-treated cohorts. The main dataset included 1,619 samples from 420 healthy controls and 595 patients across 13 cancer types; response analyses used a discovery cohort of 964 samples from 436 patients across 9 cancer types and an independent validation cohort of 361 samples from 81 patients across 5 cancer types.

**Results:**

TCRβ-inferred estimates showed that both MAIT and iNKT abundance were reduced in pretreatment cancer PBMCs compared with healthy controls, supporting systemic perturbation of unconventional T cells in cancer. Abundance estimates were also tissue dependent, with MAIT showing lower inferred abundance in tumor biopsies than in PBMCs. In ICB cohorts, baseline MAIT and iNKT estimates in PBMCs or tumors were insufficient to distinguish responders from non-responders. Although on-treatment tumor iNKT estimates showed a modest enrichment in responders in the discovery cohort, this pattern had limited standalone predictive value and was not reproduced as a robust biomarker in validation.

**Discusson:**

These findings indicate that MAIT/iNKT abundance captures cancer-associated and tissue dependent immune variation but has limited value alone for predicting ICB response, highlighting the need to incorporate functional state, spatial localization, and broader immune context.

## Introduction

Immune checkpoint blockade (ICB) has demonstrated that durable tumor control can be achieved by reinvigorating endogenous antitumor immunity (1). Yet clinical benefit remains confined to a subset of patients, even among tumors with high mutational burden, PD-L1 expression or inflamed transcriptional programs. This heterogeneity indicates that established biomarkers capture only part of the immune architecture required for therapeutic response (2, 3). A central challenge is to determine which additional immune compartments shape, accompany or constrain effective ICB.

Most studies of immunotherapy response have focused on conventional αβ T cells, particularly neoantigen-reactive CD8^+^ T cells, exhausted progenitor populations and cytotoxic effector states. This focus is supported by clinical and mechanistic studies showing that pre-existing CD8^+^ T cell infiltration, reinvigoration of exhausted-like T cells, and dynamic remodeling of tumor-reactive T cell clones are associated with response to PD-1 blockade (4–9). However, antitumor immunity is not governed by conventional T cells alone. Tumor rejection requires coordinated interactions among antigen-presenting cells, innate lymphocytes, myeloid populations, tissue-resident immune cells and systemic inflammatory cues (10–12). Immune lineages that bridge innate and adaptive immunity may therefore influence therapeutic outcome in ways that are not fully captured by conventional T cell abundance, clonality or exhaustion markers (13, 14).

MAIT cells and iNKT cells are two major unconventional T cell lineages positioned at this interface. MAIT cells recognize riboflavin-derived microbial metabolites presented by MR1 (15), whereas iNKT cells recognize lipid antigens presented by CD1d (16). Both lineages combine semi-invariant antigen recognition with rapid effector capacity, tissue enrichment and broad immunomodulatory potential (17, 18). Through inflammatory cytokine production, cytotoxic activity and crosstalk with dendritic cells, NK cells, myeloid cells and conventional T cells, MAIT and iNKT cells may influence local inflammatory tone and innate–adaptive immune coordination (19–21). These properties make them plausible contributors to the immune contexture that determines whether ICB leads to coordinated and durable tumor control (22).

The role of MAIT and iNKT cells in cancer, however, remains difficult to resolve. These cells can support inflammatory and cytotoxic responses, but they may also acquire dysfunctional, regulatory or tissue-repair-associated phenotypes in chronically inflamed tumor microenvironments (14, 23–25). MAIT cells may be influenced by MR1-dependent microbial or tumor-associated metabolic signals (23, 26), whereas iNKT cells respond to CD1d-presented lipid antigens that can be altered in cancer-associated lipid environments. Their abundance may therefore reflect multiple, potentially opposing biological processes, including tissue recruitment, antigenic stimulation, immune exhaustion and systemic inflammation. Although individual studies have linked MAIT or iNKT cells to antitumor immunity and immunotherapy outcomes (21, 24, 27–30), the robustness and generalizability of these observations across clinical cohorts remain insufficiently defined.

A major barrier to addressing this question at clinical scale has been measurement. MAIT and iNKT cells are classically defined by semi-invariant TCRα-chain usage, supported by MR1 or CD1d tetramer staining, paired αβ TCR sequencing, single-cell profiling or lineage-specific phenotyping (31–33). However, such measurements are rarely available in large immunotherapy cohorts with longitudinal sampling and mature clinical outcomes. By contrast, bulk TCRβ sequencing has been widely generated across clinical cancer studies (34–39). Although β-chain sequences were previously considered insufficient to resolve MAIT and iNKT identity because of the central role of invariant α chains, our recent study showed that artificial intelligence can learn β-chain features that enable accurate estimation of MAIT and iNKT abundance from bulk TCRβ repertoires (40). This methodological advance creates an opportunity to interrogate unconventional T cell abundance across existing clinical cohorts at a scale that was previously inaccessible.

Here, we applied this framework to systematically evaluate TCRβ-inferred MAIT and iNKT abundance across cancer and immunotherapy cohorts. We first mapped their distribution across cancer types, blood and tumor compartments, and treatment timepoints. We then tested whether these abundance estimates were associated with response to ICB in a large integrated public cohort and an independent validation cohort. By moving from technical feasibility to cross-cohort clinical evaluation, our study defines the potential and limitations of abundance-based MAIT and iNKT measurements as biomarkers of cancer immunotherapy response.

## Materials and methods

### Study design

This study aimed to evaluate the distribution and clinical relevance of TCRβ-inferred relative abundance of MAIT and iNKT cells in cancer and ICB cohorts (**Figure 1**). We analyzed curated bulk TCRβ repertoire datasets from healthy controls, patients with cancer and patients treated with ICB. MAIT and iNKT relative abundance was estimated using an AI-based TCRβ inference framework (40). The primary analyses assessed cancer-associated changes in peripheral blood, tissue compartmentalization between peripheral blood mononuclear cell (PBMC) and tumor samples, association with response to ICB and reproducibility in an independent validation cohort. All analyses were retrospective and based on de-identified public or curated datasets. Samples were analyzed at the repertoire level and, where applicable, aggregated at the patient–tissue–treatment timepoint level to reduce overrepresentation of individuals with multiple samples.

**Figure 1:**
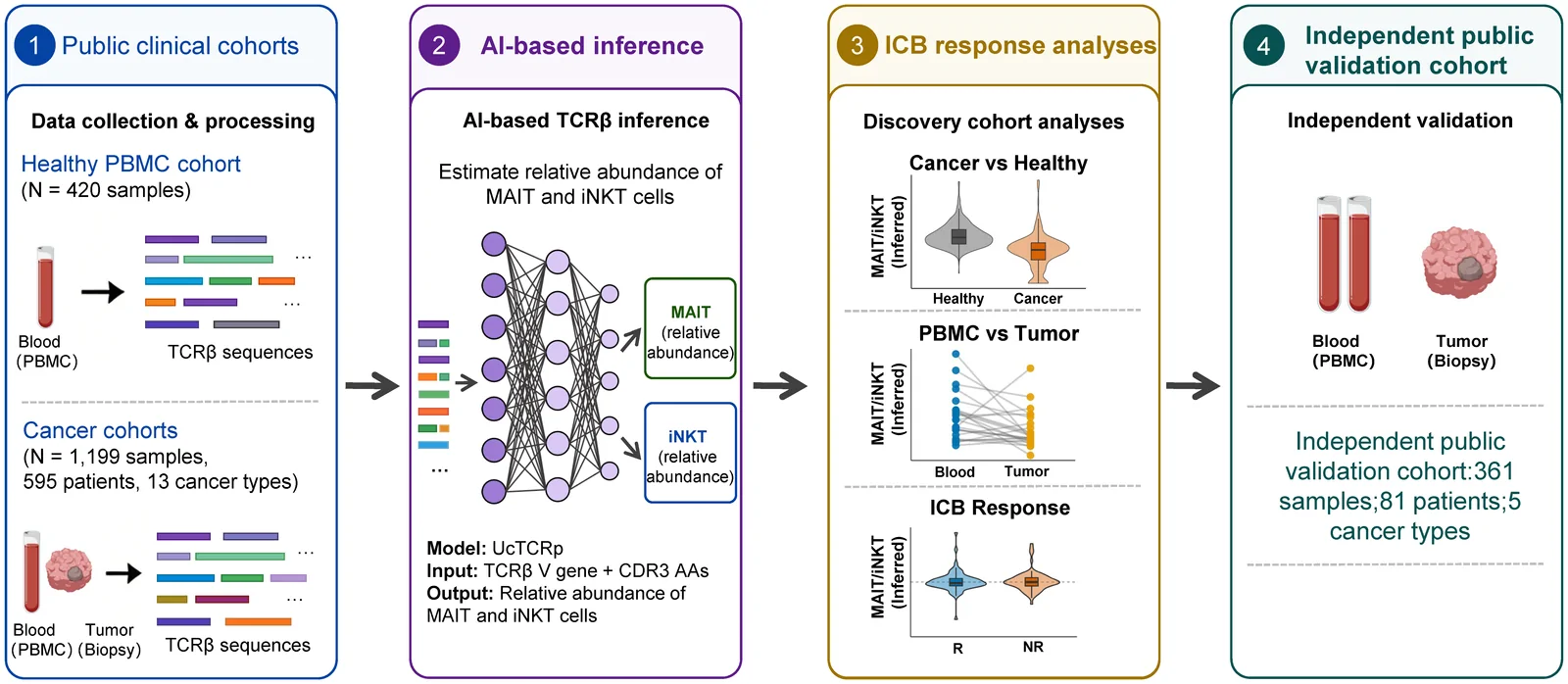
Schematic overview of the study design. Bulk TCRβ repertoires from healthy controls and patients with cancer were analyzed using a previously validated AI-based inference framework to estimate the relative abundance of MAIT and iNKT cells. The analysis included healthy control PBMC samples, cancer PBMC samples, and tumor biopsies repertoires. Downstream analyses evaluated cancer-associated changes, tissue compartmentalization, association with ICB response and independent validation.

### TCRβ repertoire datasets

The discovery dataset included bulk TCRβ repertoires from healthy controls and patients with cancer, assembled from 17 publicly available datasets (**Supplementary Table 1**). The dataset comprised 1,619 T cell repertoire samples, including 420 healthy control PBMC samples and 1,199 cancer samples from 595 patients across 13 cancer types (**Supplementary Table 2**). The cancer cohort spanned melanoma (MEL), breast cancer (BC), prostate cancer (PCa), urothelial carcinoma (UC), diffuse large B-cell lymphoma (DLBCL), non-small cell lung cancer (NSCLC), multiple myeloma (MM), basal cell carcinoma (BCC), head and neck squamous cell carcinoma (HNSCC), oral cavity squamous cell carcinoma (OSCC), mediastinal lymphoma (ML), colorectal cancer (CRC) and squamous cell carcinoma (SCC), with melanoma representing the largest subgroup. Cancer samples included PBMC, tumor biopsies and cfDNA-derived repertoires. cfDNA-derived samples were excluded from tissue-stratified cellular analyses.

For response-associated analyses, we focused on PBMC and tumor samples from patients treated with ICB and with available clinical response annotation. The response-evaluable discovery cohort included 964 PBMC or tumor TCRβ samples from 436 patients across 9 cancer types. Samples were collected before treatment or during treatment with anti-PD-1, anti-PD-L1, anti-CTLA-4 or combination ICB regimens.

An independent validation cohort was analyzed to assess reproducibility. This cohort included 361 TCRβ samples from 81 patients across five cancer types, including MEL, HNSCC, mixed solid tumors (MST), high-grade serous ovarian cancer (HGSOC) and triple-negative breast cancer (TNBC) (**Supplementary Table 3**). The validation cohort included 304 PBMC samples and 57 tumor samples collected before or during therapy.

### Estimation of MAIT and iNKT relative abundance from TCRβ repertoires

MAIT and iNKT relative abundance was inferred from bulk TCRβ repertoires using UcTCRp (40), a TCRβ-only AI framework developed to profile unconventional T cell-associated repertoire states. UcTCRp integrates V-gene context and CDR3β sequence features using a transformer-based representation pretrained on large-scale TCRβ repertoires and supervised with curated MAIT, iNKT and conventional T cell references. The framework was designed to learn lineage-informative β-chain features while reducing reliance on germline-segment shortcuts through V-matched negative sampling. UcTCRp was benchmarked across independent reference datasets to recover MAIT- and iNKT-associated β-chain patterns. Discriminative performance was assessed using receiver operating characteristic (ROC) curves and the area under the ROC curve (AUROC). The framework achieved AUROC values of 0.87 and 0.92 for distinguishing MAIT- and iNKT-associated TCRβ sequences, respectively, in independent validation datasets (40). Based on these validated sequence-level predictions, UcTCRp outputs were subsequently interpreted as TCRβ-inferred relative abundance estimates at the repertoire level. Although these sequence-based estimates are not equivalent to direct experimental quantification, they provide a scalable approach for comparing MAIT- and iNKT-associated abundance across samples and cohorts, creating an opportunity to investigate unconventional T cells in existing clinical datasets at a scale that was previously inaccessible. For visualization and reporting, inferred values were expressed on a percentage scale where appropriate.

### Sample preprocessing and filtering

Samples were filtered before downstream analysis. Sorted CD8 T cell subsets were excluded from the primary analysis to focus on bulk T cell repertoires. cfDNA-derived repertoires were retained for dataset overview but excluded from tissue-stratified cellular comparisons involving PBMC and tumor biopsies. Samples without response annotation were excluded from response-associated analyses. For the discovery ICB cohort, analyses were restricted to PBMC and tumor samples with harmonized responder or non-responder labels and treatment timepoint annotation. Treatment timepoints were categorized as pre-treatment or on-treatment according to the original study annotations. For the validation cohort, baseline samples were defined by screening blood or tumor timepoints, and all later treatment-associated samples were categorized as on-treatment.

### Patient-level aggregation

To avoid overrepresentation of patients with multiple samples within the same analytical category, samples were aggregated at the patient–tissue–treatment timepoint level. Specifically, for each patient, MAIT and iNKT relative abundance estimates were averaged within each tissue compartment and treatment timepoint. Aggregation was performed separately for PBMC and tumor samples and separately for pre-treatment and on-treatment samples. For cancer versus healthy control comparisons, pre-treatment cancer PBMC samples were compared with healthy control PBMC samples. When multiple pre-treatment PBMC samples were available from the same cancer patient, inferred relative abundance estimates were averaged at the patient level before comparison.

### Adjustment for demographic and global repertoire covariates

To account for potential demographic and repertoire-level confounding, mixed-effects models were applied separately to each analysis. For the cancer-versus-healthy comparison in **Figure 2**, linear mixed-effects models were fitted separately for inferred MAIT and iNKT relative abundance. Analyses were restricted to donors with complete information on age, sex, clonality and richness. Inferred relative abundance was converted from percentage to proportion and logit-transformed before model fitting to accommodate the bounded and right-skewed distribution of proportion data. Age, clonality, and richness were standardized to a mean of zero and a standard deviation of one, whereas sex and disease group were included as categorical variables, with female donors and healthy controls as the respective reference groups. Source dataset was included as a random intercept to account for between-dataset heterogeneity. The model was specified as:

**Figure 2:**
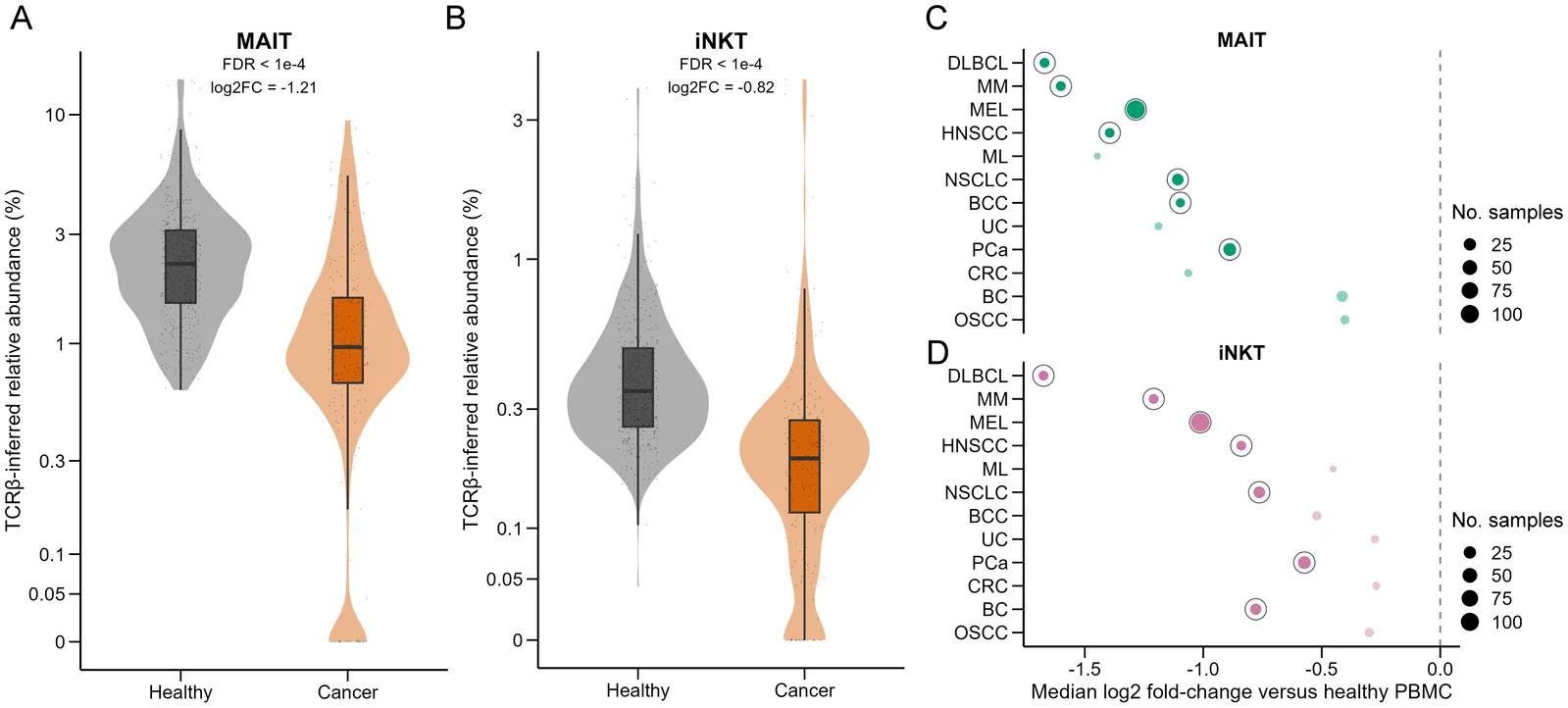
TCRβ-inferred MAIT and iNKT relative abundance is reduced in cancer PBMCs. **(A)** Comparison of TCRβ-inferred MAIT relative abundance between healthy control PBMCs and pre-treatment cancer PBMCs. **(B)** Comparison of TCRβ-inferred iNKT relative abundance between healthy control PBMCs and pre-treatment cancer PBMCs. **(C)** Cancer type-stratified effect sizes for MAIT relative abundance in cancer PBMCs compared with healthy control PBMCs. Effect sizes are shown as median log2 fold-change relative to healthy controls. **(D)** Cancer type-stratified effect sizes for iNKT relative abundance in cancer PBMCs compared with healthy control PBMCs. Effect sizes are shown as median log2 fold-change relative to healthy controls. Circles outlined in black indicate statistical significance (adjusted P < 0.05). Circle size represents the number of samples.


$$logitY=\beta_{0}+\beta_{1}Disease+\beta_{2}Age+\beta_{3}Sex+\beta_{4}Clonality+\beta_{5}Richness+b_{dataset}+\epsilon$$

where Y denotes the inferred MAIT or iNKT relative abundance for donor, β_1_ represents the adjusted difference between patients with cancer and healthy controls on the logit-transformed abundance scale, and b_dataset_ represents the dataset-specific random intercept. Separate models were fitted for MAIT and iNKT cells. Models were fitted using restricted maximum likelihood, and fixed-effect significance was assessed using Satterthwaite-approximated degrees of freedom. The resulting P values for the cancer-versus-healthy group effect were adjusted across the MAIT and iNKT analyses using the Benjamini–Hochberg procedure.

For the ICB response analyses in the discovery cohort, linear mixed-effects models were fitted separately for MAIT and iNKT relative abundance, with response status as the primary predictor and clonality and richness included as fixed-effect covariates. Both repertoire covariates were standardized before model fitting, and source dataset was included as a random intercept. Inferred relative abundance was converted to proportion and logit-transformed as described above. Models were fitted separately within each tissue and treatment time point.

### Clinical response annotation

Clinical response annotations were harmonized as responders and non-responders according to the original study definitions. Responders included patients annotated as having clinical benefit, complete response, partial response or durable response, depending on the source study. Non-responders included patients annotated as having progressive disease, no clinical benefit or non-durable response. Samples with unavailable or ambiguous response information were excluded from response-associated analyses.

### Association with ICB response

Response-associated analyses were performed separately by tissue compartment and treatment timepoint. In the discovery cohort, MAIT and iNKT relative abundance estimates were compared between responders and non-responders in four strata: pre-treatment PBMC, on-treatment PBMC, pre-treatment tumor and on-treatment tumor. Group comparisons were performed using two-sided Wilcoxon rank-sum tests. To assess standalone predictive value, single-variable logistic regression models were fitted using inferred MAIT or iNKT relative abundance as predictors of response. Model performance was summarized using McFadden pseudo-R². These models were intended to evaluate the explanatory power of abundance estimates alone rather than to develop a multivariable clinical prediction model.

### Longitudinal analysis

For patients with paired pre-treatment and on-treatment samples, longitudinal changes in inferred MAIT and iNKT relative abundance were calculated separately for PBMC and tumor compartments. Changes were analyzed as both absolute differences and log2 fold-changes:


$$\begin{array}{l} \text{Δ}=on-treatment\;estimate-pre-treament\;estimate\\ \log_{2}FC=\log_{2}\left(\frac{on-treatment\;estimate+\epsilon }{pre-treatment\;estimate+\epsilon }\right) \end{array}$$

Longitudinal changes were compared between responders and non-responders using two-sided Wilcoxon rank-sum tests. Paired pre-treatment and on-treatment values were also visualized to assess inter-patient heterogeneity.

### Statistical analysis

All statistical tests were two-sided unless otherwise specified. Continuous variables were summarized using medians and interquartile ranges. Between-group comparisons were performed using Wilcoxon rank-sum tests. Paired comparisons were performed using Wilcoxon signed-rank tests. Correlations were assessed using Spearman rank correlation. Logistic regression was used to estimate the standalone association between inferred abundance estimates and clinical response. Multiple-testing correction was performed using the Benjamini–Hochberg false discovery rate procedure within each analysis family. Statistical significance was defined as an adjusted P value below 0.05 unless otherwise stated. All analyses were performed in R version 4.3.2 and figures were generated using ggplot2.

## Results

### TCRβ-inferred MAIT- and iNKT-associated repertoire signals are reduced in cancer

We first compared pre-treatment cancer PBMC samples with healthy control PBMC samples to determine whether unconventional T cell associated repertoire signals differed between cancer and non-cancer states. After donor-level aggregation, this analysis included 638 donors, comprising 420 healthy controls and 218 patients with cancer across 12 cancer types. TCRβ-inferred MAIT relative abundance was markedly lower in cancer PBMCs than in healthy controls, with median inferred values of 0.96% and 2.23%, respectively, when expressed on a percentage scale (**Figure 2A**). iNKT relative abundance was similarly reduced, with median inferred values of 0.20% in cancer PBMCs and 0.35% in healthy controls (**Figure 2B**). The inferred values in healthy controls were broadly consistent with previously reported peripheral blood frequencies of MAIT and iNKT cells (approximately 1-10% and 0.01-1% of circulating T cells, respectively), supporting the biological plausibility of these TCRβ-based estimates (41, 42). To evaluate whether these cancer-associated differences were independent of demographic factors, we performed linear mixed-effects modeling in donors with age, sex, clonality and richness information (n = 424; 363 healthy controls and 61 patients with cancer). After adjustment, cancer status remained significantly associated with reduced TCRβ-inferred MAIT- (p < 1e-04, beta = -4.73) and iNKT-associated (p < 1e-04, beta = -4.45) repertoire signals.

Moreover, the direction of reduction was broadly consistent across cancer types, although the magnitude varied by tumor lineage and cohort size (**Figures 2C, D**; **Supplementary Figure 1**). MAIT relative abundance estimates were significantly reduced in DLBCL, MM, MEL, HNSCC, ML, and NSCLC with weaker or non-significant reductions in smaller cohorts (**Figure 2C**). iNKT relative abundance estimates showed a similar but generally smaller reduction across multiple tumor types, including DLBCL, MM, MEL, BC, NSCLC, and PCa (**Figure 1D**). Together, these findings indicate that cancer is associated with systemic reduction of TCRβ-inferred MAIT and iNKT relative abundance in peripheral blood.

### Tissue compartment differentially shapes inferred MAIT- and iNKT-associated repertoire signals

We next examined how inferred MAIT and iNKT relative abundance varied across tissue compartments in cancer. From the response-evaluable immunotherapy dataset, we identified patients with matched PBMC and tumor samples across 9 cancer types. After aggregation at the patient-tissue-treatment level, 194 analytical units from 78 patients were available for tissue- and timepoint-stratified analyses. Among patients with matched PBMC and tumor samples, MAIT relative abundance was consistently lower in tumor biopsies than in matched PBMCs both before treatment (**Figure 3A**) and on treatment (**Figure 3B**). In contrast, iNKT relative abundance showed greater inter-patient variability and did not demonstrate consistent tumor depletion (**Figures 3C, D**). Across these samples, inferred MAIT abundance ranged approximately from 0.2% to 8% in PBMCs and from 0.01% to 5% in tumors, whereas inferred iNKT abundance was generally concentrated between 0.01% and 1% in PBMCs and ranged up to approximately 1.2% in tumors. These magnitudes were broadly comparable to previously reported tissue frequencies, including MAIT-cell frequencies of approximately 2.8%-3.2% in colorectal tumors and iNKT-cell frequencies of approximately 0.27% among tumor-infiltrating lymphocytes in intrahepatic malignancies (43–45). Cancer-type-stratified analyses showed a consistent reduction of inferred MAIT-associated signals in tumors across HNSCC, OSCC, PCa, and UC, whereas iNKT-associated signals showed no consistent pattern (**Supplementary Figure 2**). Due to limited sample sizes within individual cancer types, statistically significant differences were observed only in the OSCC subgroup.

**Figure 3:**
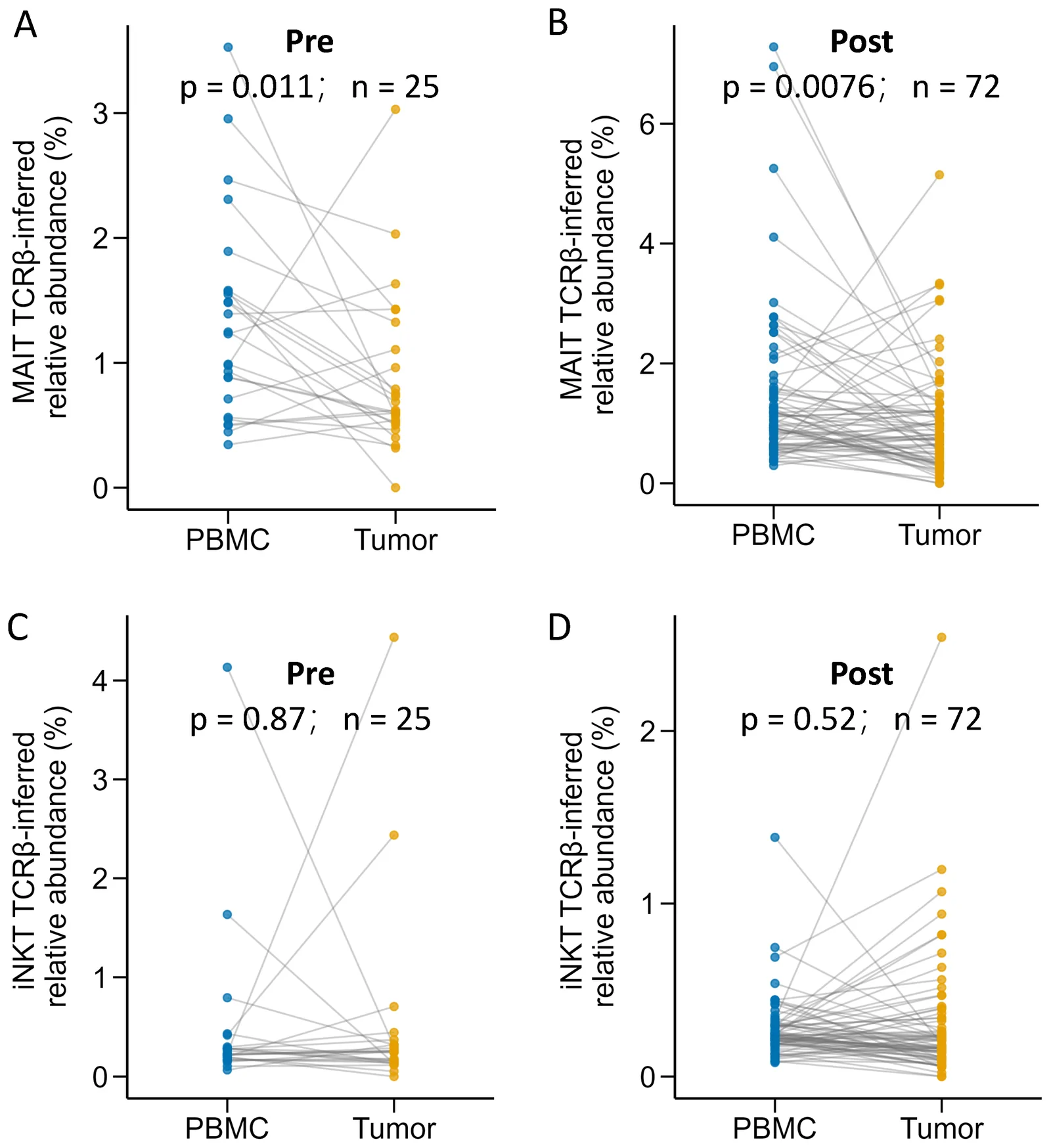
Tissue compartment shapes inferred MAIT and iNKT-associated repertoire signals. **(A–D)** Paired PBMC–tumor comparisons of inferred MAIT- **(A, B)** and iNKT-associated **(C, D)** relative abundance before treatment **(A, C)** and during treatment **(B, D)**. Lines connect matched samples from the same patient.

### Inferred MAIT- and iNKT-associated repertoire signals show limited association with ICB response in the discovery cohort

We next evaluated whether estimated MAIT or iNKT relative abundance was associated with response to ICB. The discovery immunotherapy cohort included 964 response-evaluable PBMC or tumor TCRβ samples from 436 patients across 9 cancer types. After patient–tissue–treatment aggregation, the analysis included 162 pre-treatment PBMC units, 237 post-treatment PBMC units, 85 pre-treatment tumor units and 255 post-treatment tumor units (**Figure 4A**).

**Figure 4:**
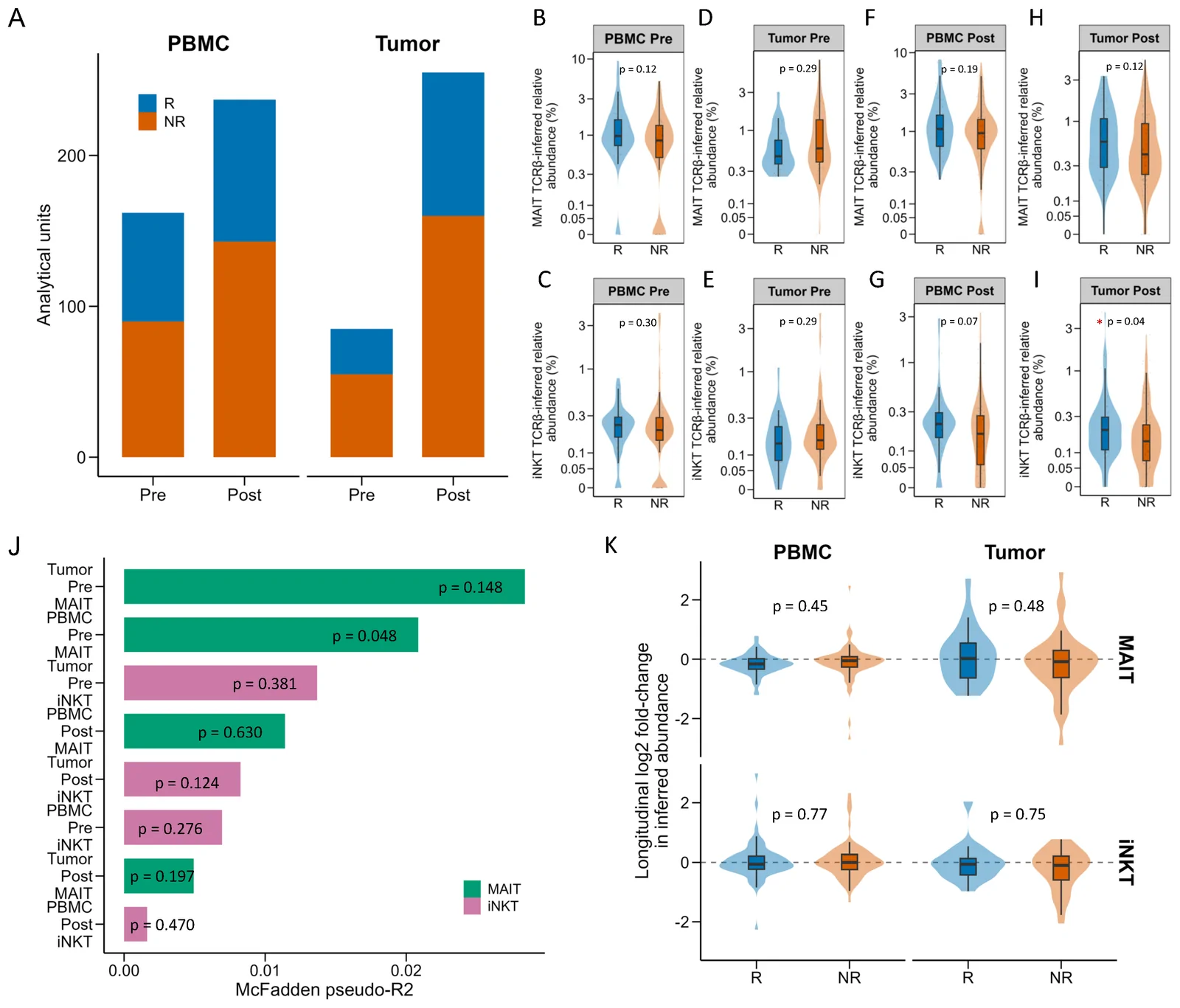
Estimated MAIT and iNKT abundance has limited association with ICB response in the discovery cohort. **(A)** Composition of the discovery ICB cohort. Response-evaluable PBMC and tumor TCRβ samples were aggregated at the patient–tissue–treatment level and analyzed according to tissue compartment, treatment timepoint and clinical response. **(B, C)** Baseline PBMC MAIT and iNKT relative abundance estimates in responders and non-responders. **(D, E)** Baseline tumor MAIT and iNKT relative abundance estimates in responders and non-responders. **(F, G)** On-treatment PBMC MAIT and iNKT relative abundance estimates in responders and non-responders. **(H, I)** On-treatment tumor MAIT and iNKT relative abundance estimates in responders and non-responders. **(J)** Standalone predictive value of MAIT and iNKT relative abundance estimates across tissue and timepoint strata, assessed using single-variable logistic regression models. Model performance is shown as McFadden pseudo-R², where higher values indicate greater improvement over a null model and stronger standalone predictive ability. **(K)** Longitudinal changes in MAIT and iNKT relative abundance from baseline to on-treatment sampling in responders and non-responders, analyzed separately in PBMC and tumor compartments.

At baseline, estimated MAIT and iNKT relative abundance did not robustly distinguish responders from non-responders. In pre-treatment PBMCs, MAIT estimates were numerically higher in responders than in non-responders, but the effect size was small relative to inter-patient variability and did not remain significant after multiple-testing correction (**Figure 4B**). Pre-treatment PBMC iNKT estimates were also largely overlapping between response groups (**Figure 4C**). In pre-treatment tumor biopsies, neither MAIT nor iNKT estimates separated responders from non-responders (**Figures 4D, E**).

Post-treatment samples showed modest response-associated trends. Post-treatment PBMC and tumor MAIT estimates showed a weaker numerical increase in responders (**Figures 4F, H**). In PBMC collected during therapy, iNKT relative abundance was numerically higher in responders than in non-responders, but this association did not remain significant after correction (**Figure 4G**). In post-treatment tumor biopsies, iNKT relative abundance was higher in responders than in non-responders, with median inferred values of 0.21% and 0.15%, respectively (**Figure 4I**), but this enrichment was not consistently observed across tumor types (**Supplementary Figure 3**).

Because responders and non-responders also differed in broader TCR repertoire features after treatment (**Supplementary Figure 4**), we further evaluated MAIT/iNKT-associated signals using mixed-effects models adjusted for richness and clonality. After adjustment, the responder-associated increase in tumor iNKT-associated signal was attenuated and no longer statistically significant (p = 0.26). Similar results were observed in treatment-stratified analyses, including the combination-therapy subgroup, where the unadjusted tumor iNKT association was not retained after adjustment (**Supplementary Figure 5**).

Morevoer, single-variable logistic regression models using MAIT or iNKT relative abundance explained only a small fraction of response variability across tissue and timepoint strata, as measured by McFadden pseudo-R² (**Figure 4J**; values near zero indicate limited improvement over a null model). Even for post-treatment tumor iNKT abundance, the model showed low explanatory power, indicating limited individual-level predictive utility (**Figure 4J**).

We further analyzed a subset of patients with paired pre-treatment and post-treatment samples (n = 204). Longitudinal changes in MAIT and iNKT relative abundance were heterogeneous across individuals and did not identify a consistent response-specific pattern (**Figure 4K**). In PBMC, MAIT changes showed a nominal difference between responders and non-responders, but this did not remain significant after correction. PBMC iNKT changes were not associated with response, and neither MAIT nor iNKT changes in tumor tissue showed a reproducible response-associated pattern.

These findings indicate that estimated MAIT and iNKT abundance is not a robust baseline biomarker of ICB response. Post-treatment tumor iNKT abundance may capture a weak responder-enriched signal in the discovery cohort, but the effect size and standalone predictive value are limited.

### Independent validation confirms limited predictive value of inferred MAIT and iNKT-associated repertoire signals

To test reproducibility, we analyzed an independent validation cohort comprising 361 TCRβ samples from 81 patients across five cancer types. The validation cohort included 304 PBMC samples and 57 tumor samples collected before or during treatment. After aggregation at the patient–tissue–timepoint level, 200 analytical units were available for validation analysis (**Figure 5A**).

**Figure 5:**
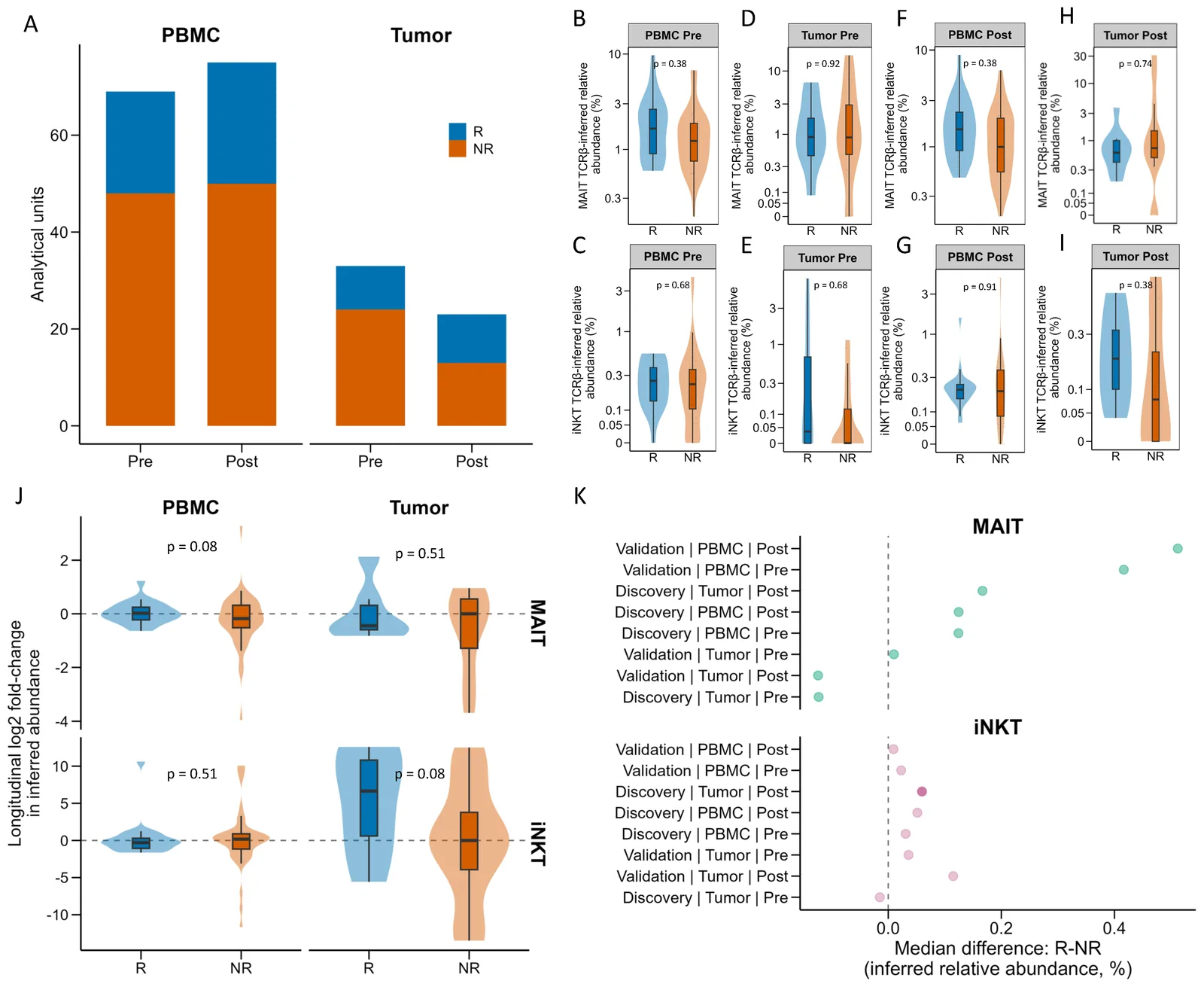
Independent validation confirms limited predictive value of inferred MAIT and iNKT abundance. **(A)** Overview of the independent validation cohort. The cohort included PBMC and tumor TCRβ samples collected before and during therapy from patients across five cancer types. Samples were aggregated at the patient–tissue–timepoint level for response-associated analyses. **(B, C)** Baseline PBMC MAIT and iNKT relative abundance estimates in responders and non-responders in the validation cohort. **(D, E)** Baseline tumor MAIT and iNKT relative abundance estimates in responders and non-responders in the validation cohort. **(F, G)** On-treatment PBMC MAIT and iNKT relative abundance estimates in responders and non-responders in the validation cohort. **(H, I)** On-treatment tumor MAIT and iNKT relative abundance estimates in responders and non-responders in the validation cohort. **(J)** Longitudinal changes in MAIT and iNKT relative abundance from baseline to on-treatment sampling in the validation cohort, analyzed separately in PBMC and tumor compartments. **(K)** Summary of response-associated effect sizes across the discovery and validation cohorts. Effect sizes are shown for MAIT and iNKT estimates across tissue compartments and treatment timepoints. Positive values indicate higher inferred relative abundance in responders, whereas negative values indicate higher inferred relative abundance in non-responders.

Consistent with the discovery cohort, baseline MAIT and iNKT relative abundance estimates did not significantly distinguish responders from non-responders. In baseline PBMC samples, MAIT estimates were numerically higher in responders than in non-responders, with median inferred values of 1.64% and 1.23%, respectively, but this difference was not significant after multiple-testing correction (**Figure 5B**). Baseline PBMC iNKT estimates were similar between response groups (**Figure 5C**). In baseline tumor samples, both MAIT and iNKT estimates were largely overlapping between responders and non-responders (**Figures 5D, E**).

Post-treatment samples showed no reproducible response-associated difference. In PBMC, MAIT estimates were again numerically higher in responders but did not reach significance after correction, whereas iNKT estimates were nearly identical between response groups (**Figures 5F, G**). In tumor biopsies collected during therapy, MAIT estimates in on-treatment tumor samples were not higher in responders (**Figure 5H**), iNKT estimates showed a modest numerical increase in responders, but this trend was not statistically significant (**Figure 5I**). The validation cohort did not show consistent cancer type-specific response associations for inferred MAIT or iNKT abundance (**Supplementary Figure 6**).

Longitudinal analyses confirmed the absence of a stable response-specific pattern. Among patients with paired pre-treatment and post-treatment samples, changes in MAIT and iNKT relative abundance were heterogeneous in both PBMC and tumor compartments. Neither absolute changes nor log2 fold-changes differed significantly between responders and non-responders after correction, although responders tended to show greater increases in PBMC MAIT and tumor iNKT abundance than non-responders (**Figure 5J**).

Finally, we summarized response-associated effect sizes across discovery and validation cohorts. The discovery cohort showed a modest responder-enriched signal for on-treatment tumor iNKT abundance, but this pattern was not reproduced as a robust effect in the validation cohort (**Figure 5K**). Across most tissue, timepoint and cell-type strata, effect sizes were small and confidence intervals crossed the null.

Together, the validation cohort confirmed that TCRβ-inferred MAIT and iNKT relative abundance does not robustly stratify patients by ICB response. Across discovery and validation cohorts, these estimates capture measurable cancer-associated and tissue-dependent immune variation, but abundance alone is insufficient to define the clinical relevance of MAIT and iNKT cells in cancer immunotherapy.

## Discussion

In this study, we used lineage-associated TCRβ repertoire signals to systematically examine MAIT and iNKT cells across cancer and immunotherapy cohorts. We found that both populations were broadly perturbed in cancer, with reduced peripheral blood abundance across multiple tumor types. This systemic depletion was not accompanied by consistent enrichment in tumor tissue and showed features distinct from global repertoire variation. However, despite their biological plausibility as modulators of antitumor immunity, MAIT and iNKT abundance did not reproducibly associate with response to ICB across integrated and independent validation cohorts. These findings suggest that unconventional T cell perturbation is a detectable feature of cancer, but that frequency alone is insufficient to capture its clinical relevance in immunotherapy.

The observation that MAIT and iNKT repertoire signals are reduced in the blood of patients with cancer supports the view that the unconventional T cell compartment is broadly remodeled during malignancy (44–47). These cells are enriched at barrier and tissue sites, respond rapidly to inflammatory cues, and integrate microbial, metabolic and lipid antigen signals with innate-adaptive immune crosstalk (48, 49). Their depletion in blood may therefore reflect multiple non-mutually exclusive processes, including altered homeostasis, chronic activation, tissue redistribution, metabolic stress or phenotypic remodeling (44, 45, 47). Importantly, our data do not support a simple model in which peripheral depletion is explained by uniform recruitment into sampled tumor lesions. Instead, the low and heterogeneous detection of MAIT and iNKT signals in tumor biopsies indicates that cancer-associated perturbation of these lineages is likely shaped by tissue context, tumor type and host-level immune state (50).

The lack of reproducible association with ICB response has several implications. First, it argues against using MAIT or iNKT frequency alone as a standalone biomarker for patient stratification. In the integrated cohort, baseline frequencies in blood and tumor were largely overlapping between responders and non-responders, on-treatment differences were modest and inconsistent, and longitudinal changes did not show a stable response-specific pattern. These observations were reproduced in an independent validation cohort. Second, the data suggest that the role of unconventional T cells in cancer immunotherapy, if present, is unlikely to be encoded primarily by their abundance. This is consistent with the biology of MAIT and iNKT cells, whose effects can vary according to activation state, cytokine polarization, antigenic context, tissue localization and interactions with antigen-presenting, myeloid and conventional lymphoid compartments (48, 49).

This distinction between abundance and function is central to interpreting our findings. MAIT and iNKT cells can exert cytotoxic, inflammatory, regulatory or tissue-repair-associated functions depending on context. A numerical estimate derived from bulk TCRβ data cannot distinguish an IFN-γ-producing cytotoxic MAIT population from an IL-17-skewed, exhausted or regulatory-like state. Similarly, it cannot resolve whether iNKT-associated signals reflect cells positioned in productive antigen-presenting niches or cells excluded from relevant tumor-immune interfaces. The absence of a frequency-response association should therefore not be interpreted as evidence that MAIT and iNKT cells are irrelevant to cancer immunity. Rather, it indicates that their clinically relevant biology is not reducible to abundance alone.

Beyond these biological considerations, an additional technical consideration is that the MAIT and iNKT estimates in this study were inferred from TCRβ repertoire features using an AI-based framework rather than directly measured at the cellular level. Although UcTCRp demonstrated robust discriminatory performance in independent validation datasets, model calibration and inference performance may be influenced by sample size, cohort heterogeneity, and data quality. Therefore, these values should be interpreted as relative lineage-associated repertoire signals rather than direct measurements of cellular abundance.

Future studies should therefore move beyond repertoire signals. Single-cell multi-omic profiling with paired TCRαβ sequencing, MR1 and CD1d tetramer staining, spatial transcriptomics and functional perturbation assays will be needed to define which MAIT and iNKT states are present in tumors, where they localize, how they interact with antigen-presenting and effector cells, and whether they are modulated by ICB. Longitudinal studies that integrate blood, tumor and tumor- or microbiome-associated antigenic context may be particularly informative, as emerging studies have begun to identify tumor-associated CD1d ligands and MR1-presented metabolites, and to show that tumor-infiltrating MAIT or MR1-restricted T cells can reflect local antigenic stimulation (26, 51). Such approaches may reveal response-associated functional states that are invisible to frequency-based repertoire analysis.

Collectively, our findings show that TCRβ-inferred MAIT and iNKT relative abundance captures systemic cancer-associated perturbation and tissue-dependent immune variation, but does not provide a robust frequency-based biomarker of ICB response. This defines an important boundary for repertoire-based biomarker discovery: inferred abundance can reveal immune remodeling, but may fail to capture the functional circuitry that determines therapeutic benefit. For unconventional T cells, the clinically relevant unit is likely not abundance alone, but state, location and immune context.

## Data Availability

The original contributions presented in the study are included in the article/**Supplementary Material**. Further inquiries can be directed to the corresponding authors.
