# Supplementary material for: Systematic evaluation of MAIT and iNKT abundance as checkpoint blockade biomarkers
Source: Front Immunol. 2026 Sep 15;17:1917317. doi: 10.3389/fimmu.2026.1917317 (PMC13619815; doi:10.3389/fimmu.2026.1917317)
Supplement: Supplementary file 1 [file DataSheet1.pdf]

Supplementary Materials for

Systematic evaluation of MAIT and iNKT abundance as checkpoint blockade biomarkers

Jian Zhang *et al*.

Corresponding author: Jian Zhang, jian_zhang@tju.edu.cn (J.Z.)

The PDF file includes:

- Supplementary Figures 1 to 6

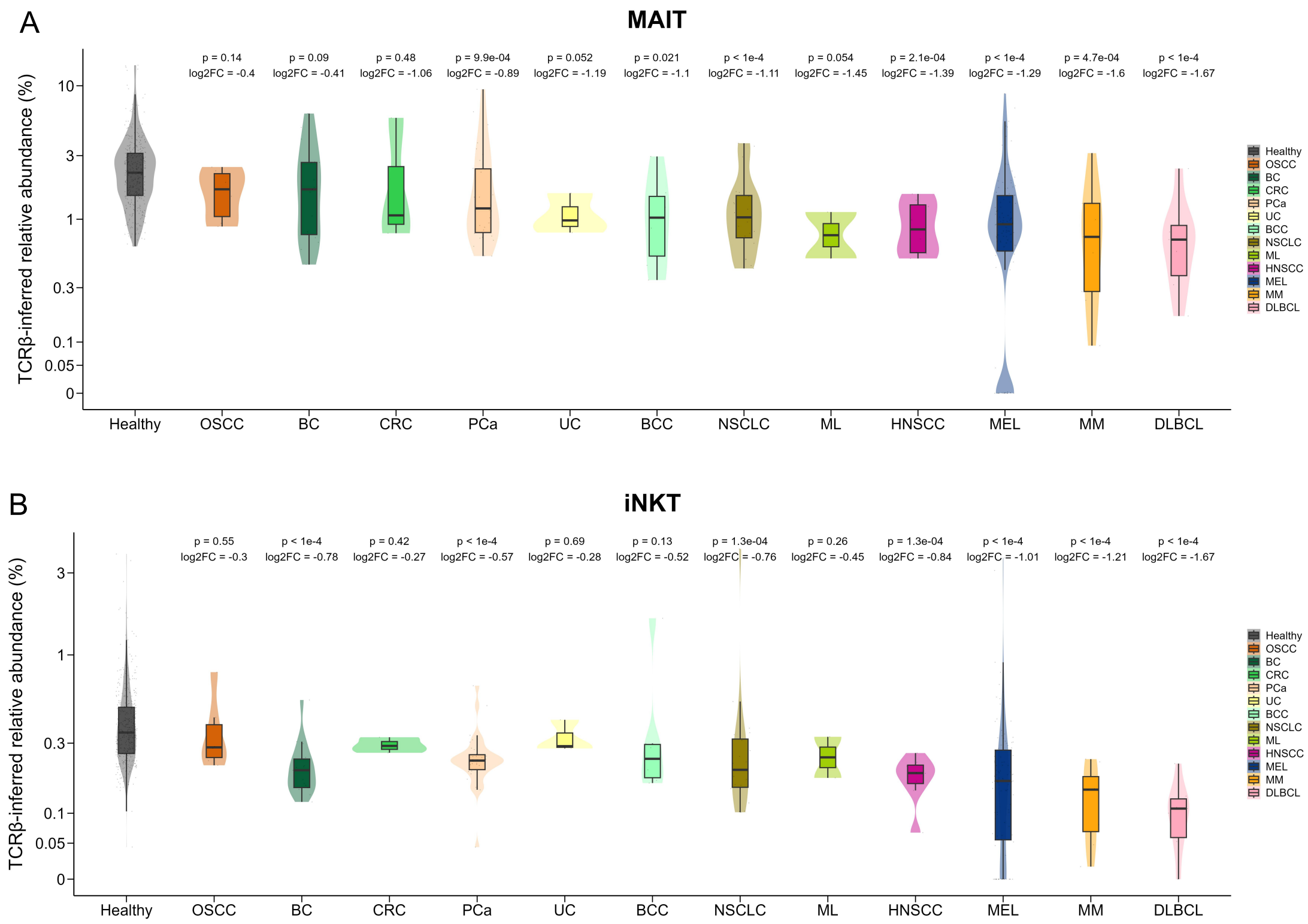

Supplementary Figure 1. Cancer-type-specific healthy vs cancer comparisons. Comparison of TCRβ-inferred MAIT (A) and iNKT (B) cell relative abundance between healthy control and individual cancer types in PBMCs. Box plots show the median, interquartile range and whiskers extending to 1.5× the interquartile range. Each point represents one sample-level estimate as indicated in the Methods. Statistical comparisons between healthy and cancer PBMCs were performed using two-sided Wilcoxon rank-sum tests. Multiple-testing correction across cancer type-stratified comparisons was performed using the Benjamini–Hochberg method.

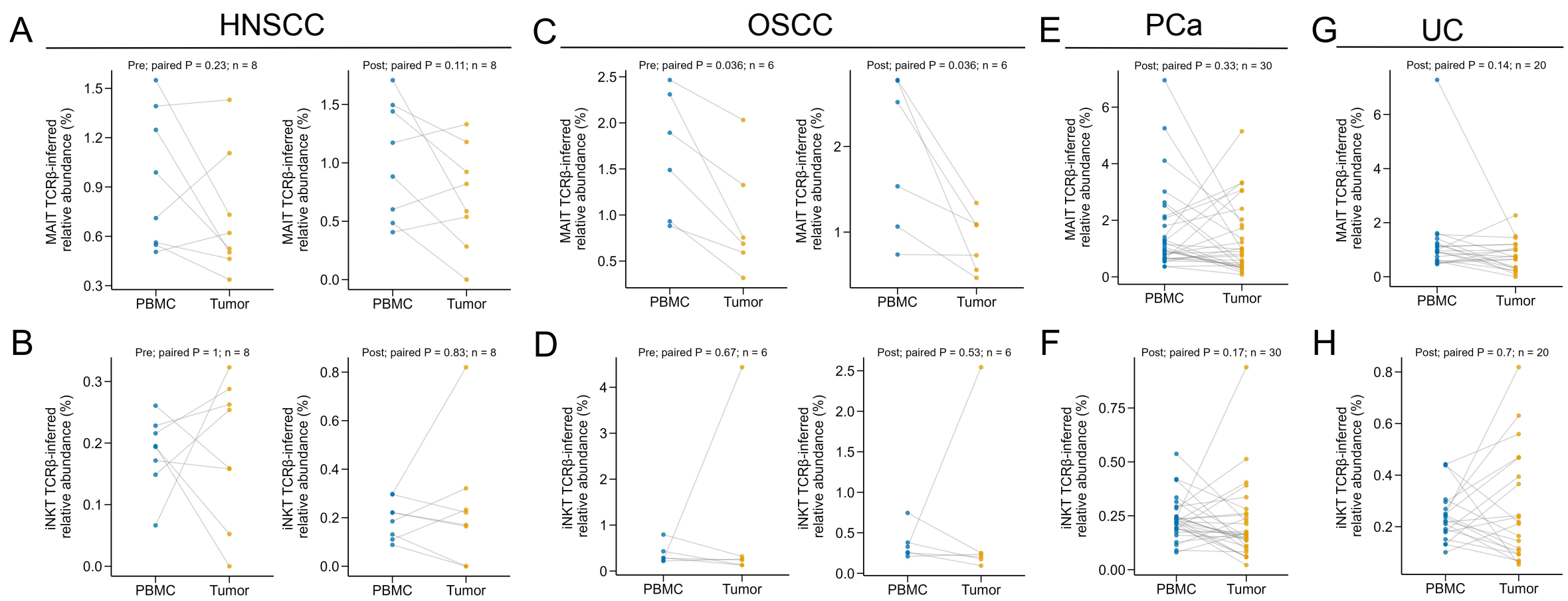

Supplementary Figure 2. Cancer type-stratified paired comparison of TCRβ-inferred MAIT and iNKT relative abundance between PBMC and tumor samples. (A-B) Paired PBMC-tumor comparisons of TCRβ-inferred MAIT (A) and iNKT (B) relative abundance in patients with HNSCC, shown separately for pre- and post-treatment samples. (C-D) Corresponding paired comparisons of MAIT (C) and iNKT (D) relative abundance in patients with OSCC, shown separately for pre- and post-treatment samples. (E-F) Paired comparisons of MAIT (E) and iNKT (F) relative abundance in post-treatment samples from patients with PCa. (G-H) Paired comparisons of MAIT (G) and iNKT (H) relative abundance in post-treatment samples from patients with UC.

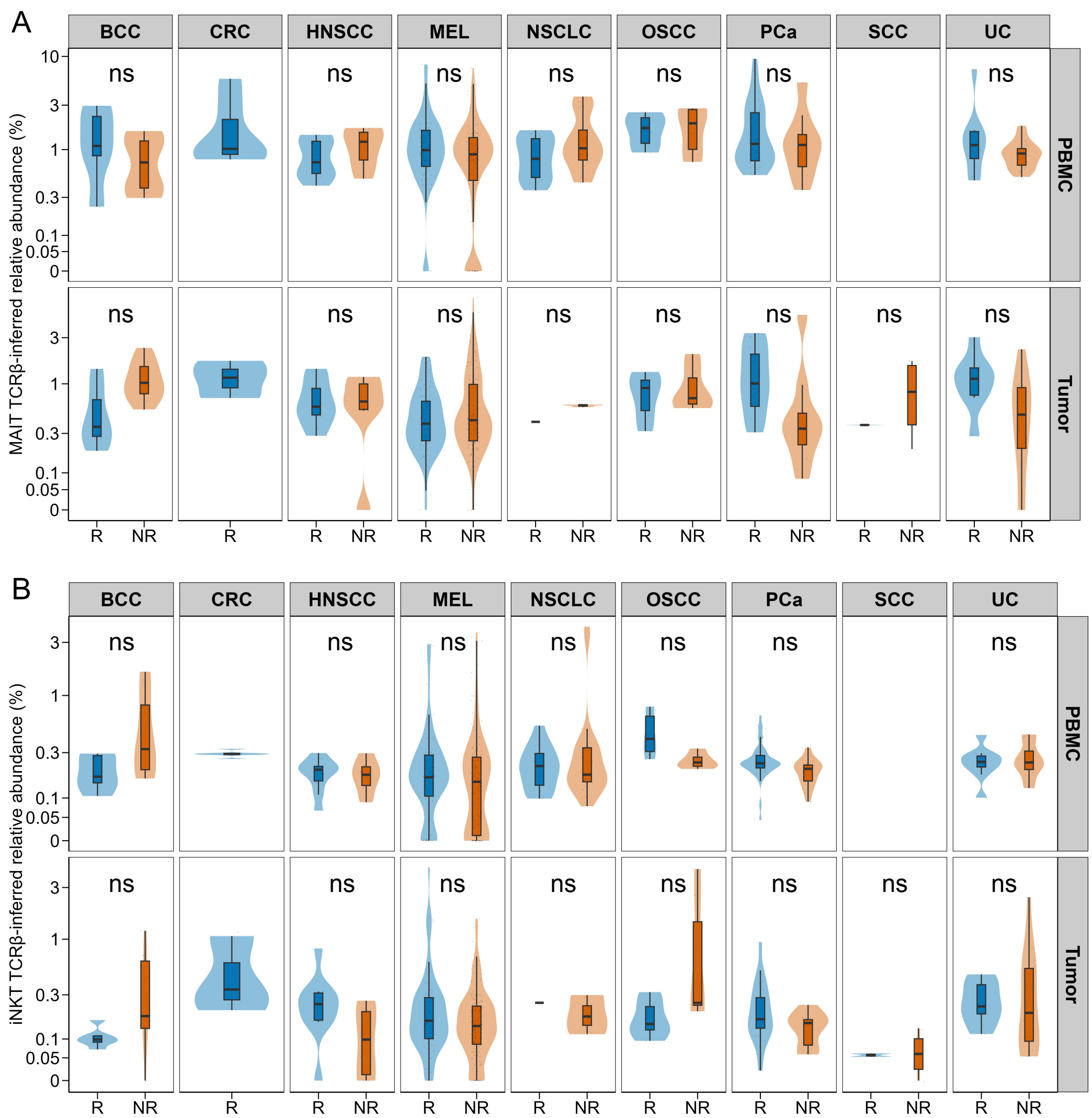

Supplementary Figure 3. Discovery cohort response comparisons stratified by cancer type. Distribution of TCRβ-inferred MAIT (A) and iNKT (B) cell relative abundance in PBMC and tumor samples, compared between responders and non-responders in discovery cohort. Samples were aggregated at the patient-tissue-response level for analysis.

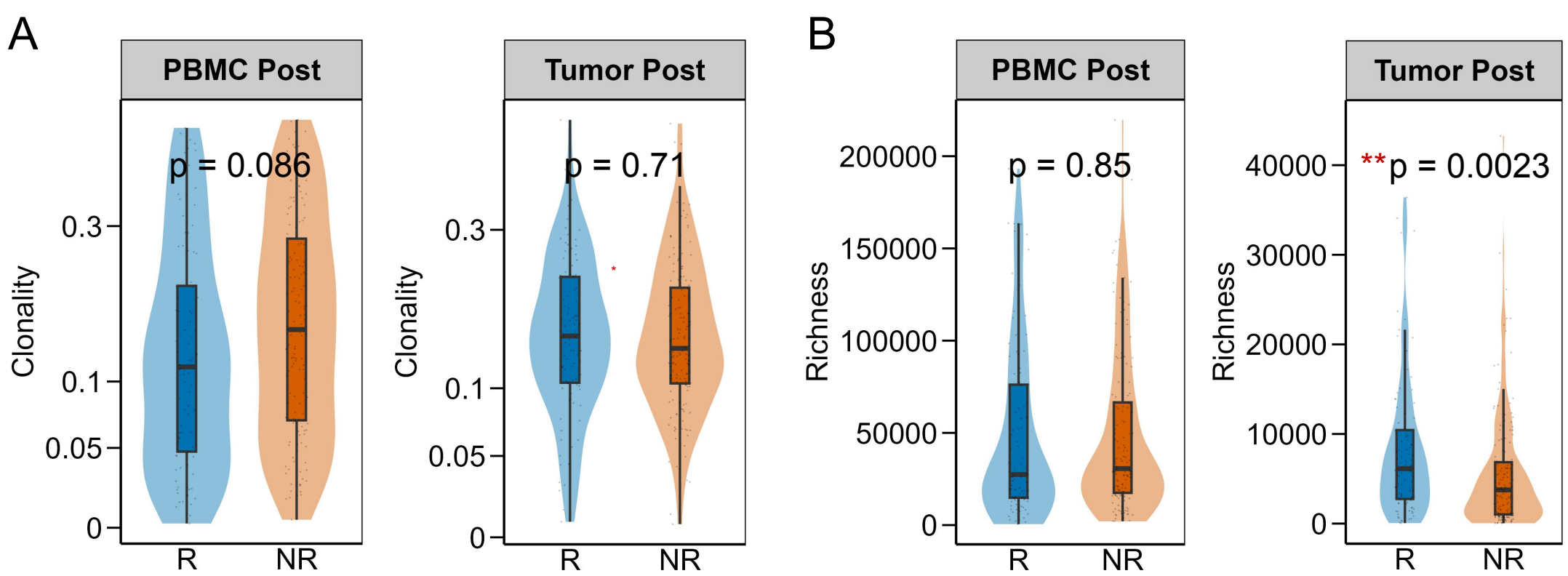

Supplementary Figure 4. Comparison of repertoire characteristics between responders and non-responders after treatment. Clonality (A) and richness (B) in post-treatment PBMC and tumor samples from responders and non-responders.

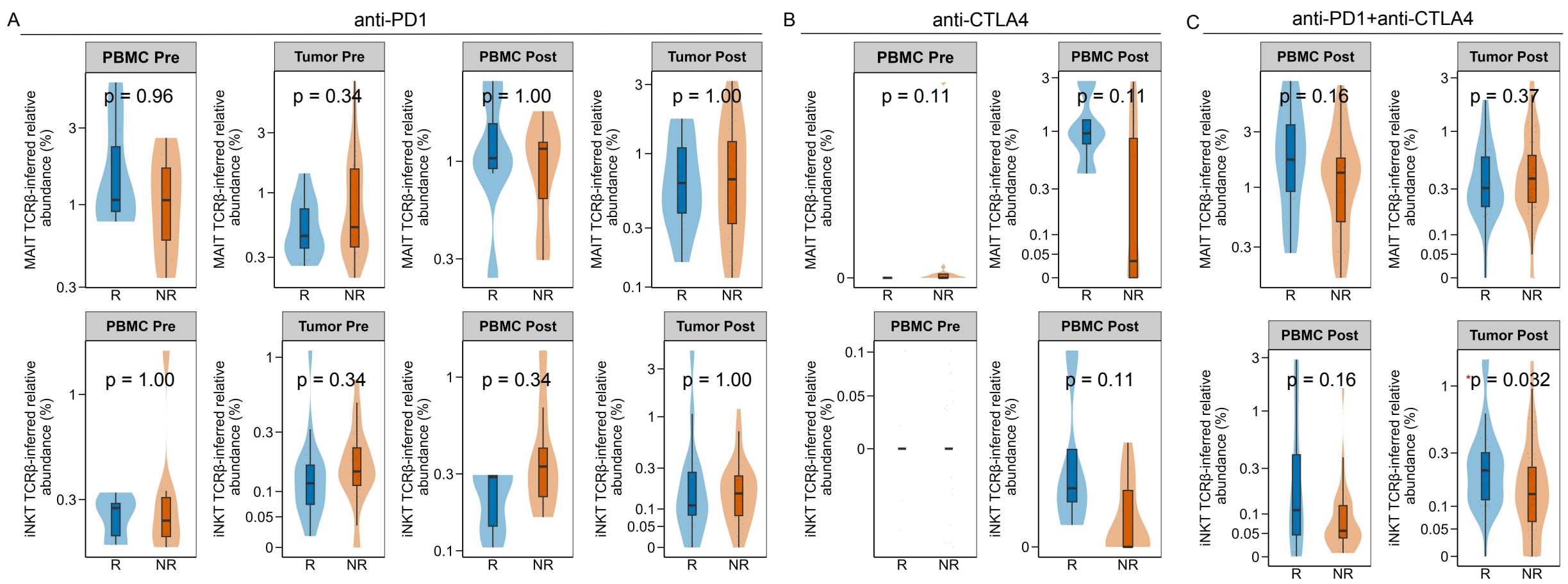

Supplementary Figure 5. Treatment-regimen-stratified associations between TCRβ-inferred MAIT and iNKT relative abundance and ICB response after adjustment of tumor iNKT estimates for global repertoire properties. Comparisons of TCRβ-inferred MAIT and iNKT relative abundance between responders and non-responders were performed separately for patients receiving anti-PD-1 therapy (A), anti-CTLA-4 therapy (B), or combined anti-PD-1 + anti-CTLA-4 therapy (C), stratified by tissue compartment and treatment time point where data were available.

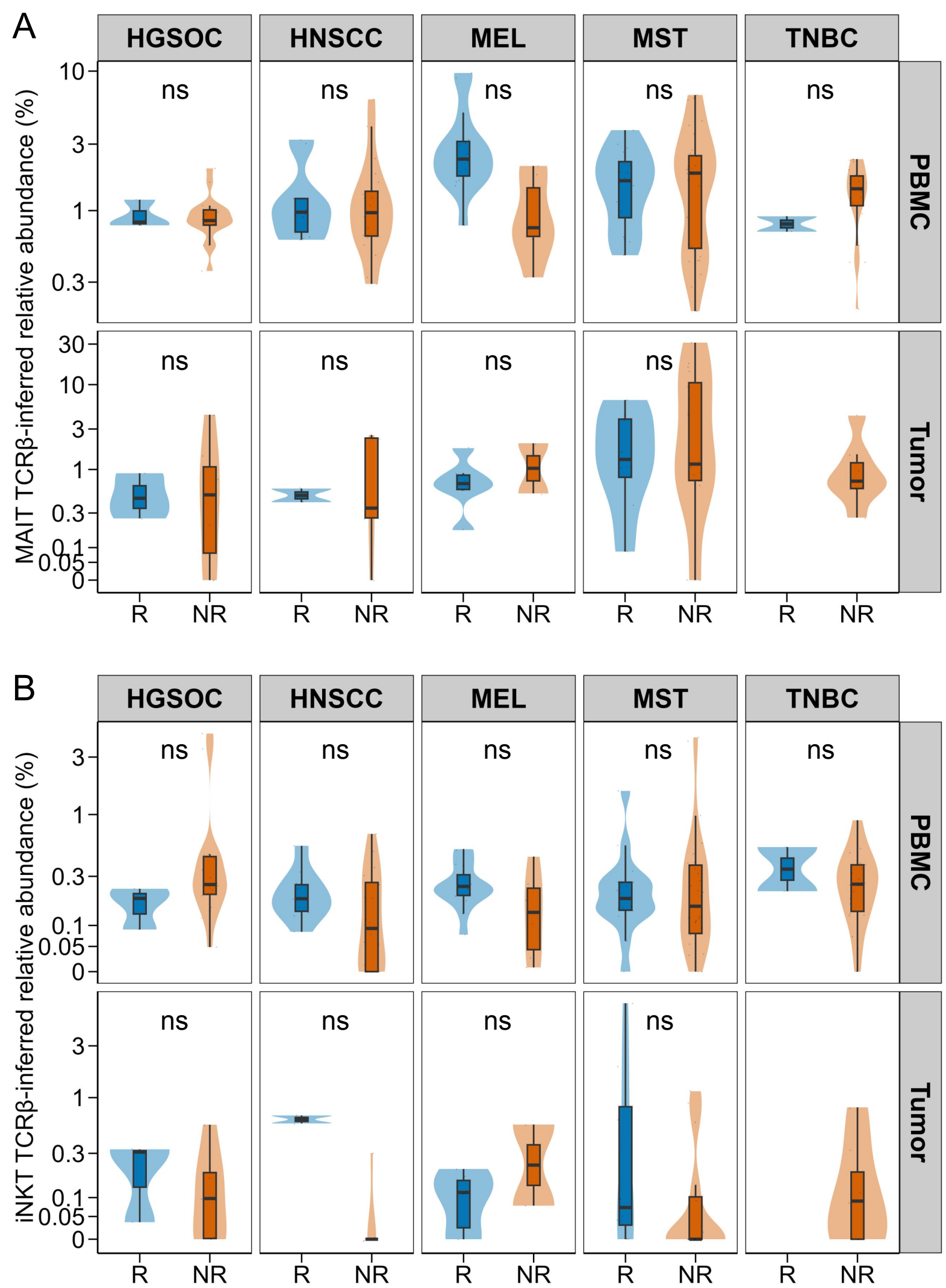

Supplementary Figure 6. The independent validation cohort response comparisons stratified by cancer type after adjustment of tumor iNKT estimates for global repertoire properties. Distribution of TCRβ-inferred MAIT (A) and iNKT (B) cell relative abundance in PBMC and tumor samples, compared between responders and non-responders in the independent validation cohort. Samples were aggregated at the patient–tissue–response level for analysis.
